# Validating Morphometrics with DNA Barcoding to Reliably Separate Three Cryptic Species of *Bombus* Cresson (Hymenoptera: Apidae)

**DOI:** 10.3390/insects11100669

**Published:** 2020-09-30

**Authors:** Joan Milam, Dennis E. Johnson, Jeremy C. Andersen, Aliza B. Fassler, Desiree L. Narango, Joseph S. Elkinton

**Affiliations:** 1 Department of Environmental Conservation, University of Massachusetts Amherst, 160 Holdsworth Way, Amherst, MA 01003, USA; jcandersen@umass.edu (J.C.A.); alizamfassler@gmail.com (A.B.F.); dnarango@gmail.com (D.L.N.); elkinton@ent.umass.edu (J.S.E.); 2 Private Practice, Eau Claire, WI 54701, USA; dermjohn@aol.com

**Keywords:** Apoidea, Bombini, malar ratio, *Bombus vagans*, *Bombus sandersoni*, *Bombus perplexus*

## Abstract

**Simple Summary:**

Evidence of bumble bee population declines has led to an increase in conservation efforts to protect these important pollinators. However, effective conservation requires accurate species identification. We provide quantitative methods to accurately identify three cryptic species of bumble bees using morphometric measurements of the cheek length and width, and antennal segments. We validated the accuracy of our methods with DNA analysis. We predicted that these methods would reliably identify both the queens and worker bees of *Bombus vagans* and *B. sandersoni*. We expanded these methods to include an uncommon form of *Bombus perplexus* with all light hair on its thorax, rather than the more common light on top and dark below, that can mistakenly be identified as *B. vagans* or *B. sandersoni*. Although the species we consider here, *Bombus vagans*, *B. sandersoni* and *B. perplexus*, are not currently listed as species of concern in North America, there is uncertainty of their population status, some of which is due to difficulty in species identification, which we have resolved. Recent history informs us that some bumble bee species experience rapid declines within a few decades. Our methods to correctly identify these cryptic species is key to monitoring their status and population trends.

**Abstract:**

Despite their large size and striking markings, the identification of bumble bees (*Bombus* spp.) is surprisingly difficult. This is particularly true for three North American sympatric species in the subgenus *Pyrobombus* that are often misidentified: *B. sandersoni* Franklin, *B. vagans* Smith *B. perplexus* Cresson. Traditionally, the identification of these cryptic species was based on observations of differences in hair coloration and pattern and qualitative comparisons of morphological characters including malar length. Unfortunately, these characteristics do not reliably separate these species. We present quantitative morphometric methods to separate these species based on the malar length to width ratio (MRL) and the ratios of the malar length to flagellar segments 1 (MR1) and 3 (MR3) for queens and workers, and validated our determinations based on DNA barcoding. All three measurements discriminated queens of *B. sandersoni* and *B. vagans* with 100% accuracy. For workers, we achieved 99% accuracy by combining both MR1 and MR3 measurements, and 100% accuracy differentiating workers using MRL. Moreover, measurements were highly repeatable within and among both experienced and inexperienced observers. Our results, validated by genetic evidence, demonstrate that malar measurements provide accurate identifications of *B. vagans* and *B. sandersoni*. There was considerable overlap in the measurements between *B. perplexus* and *B. sandersoni*. However, these species can usually be reliably separated by combining malar ratio measurements with other morphological features like hair color. The ability to identify bumble bees is key to monitoring the status and trends of their populations, and the methods we present here advance these efforts.

## 1. Introduction

Recent reports of major declines in global insect populations are cause for concern [1,2,3,4]. While declines are occurring in nearly all groups of insects, severe declines have been noted in wild bee populations [5], particularly bumble bees *Bombus* Cresson (Hymenoptera: Apidae) since the mid-twentieth century [6,7,8,9,10,11,12,13,14]. These declines have been attributed to a wide range of factors including habitat loss and fragmentation, climate change, intensified use of pesticides in agriculture, loss of floral resources, disease, and invasive species [6,7,8,9,10,15,16]. Evident declines in the range and abundance in bumble bee populations have encouraged increased research efforts to document distribution, population status and trends, extinction risks, and best management practices [5,12,14,17,18,19].

Unfortunately, despite being large-bodied with conspicuous hair coloration and patterns, many bumble bee species are deceptively challenging to identify [20,21,22,23], particularly because there are few helpful characters for separating species [24]. In North America, several identification guides exist for bumble bees [23,25,26,27], however, there are still challenges to the identification of some similarly colored cryptic species. Hair coloration is an important field characteristic for identification, yet it has been widely acknowledged that accurate identifications based on hair coloration are complicated by chromatic variability within species and convergent evolution between species [23,28,29,30]. For example, Franklin [25] confessed that he had “much difficulty in describing the colors exhibited by the pile of the various species.” Therefore, errors in identification may result without careful examination of morphological characters [28,31,32].

Three commonly misidentified sympatric species of bumble bee with similar hair coloration and patterns in the subgenus *Pyrobombus* Dalla Torre, are *B. vagans* (Half-Black Bumble Bee), *B. sandersoni* (Sanderson Bumble Bee) and *B. perplexus* (Confusing Bumble Bee). While hair color has frequently been used in bumble bee identification, there exists considerable variation in hair color patterns both within and among castes of *B. sandersonii*, *B. vagans* and *B. perplexus* [23,25,27], making reliance on this character alone insufficient. Although *B. perplexus* typically has extensive black hair on the lower half of the pleura [25,27], it has a less common light colored form that has the sides of the thorax entirely yellow to the base of the legs that is often confused with the prior two species [23]. Franklin [25] stated that light form females of *B. perplexus* were regularly confused as *B. vagans*.

In addition to their morphologic similarities, these species share a confusing history that is complicated by the fact that important early taxonomic work on this group was conducted solely on spring-caught queens and males [25,28]. Franklin initially described *B. sandersoni* as a subspecies of *B. vagans* noting slight differences in their malar space. Frison [33] did not agree with Franklin’s determinations that *B. sandersoni* was a subspecies of *B. vagans* and instead suggested that *B. sandersoni* was actually a color variety of *B. frigidus* Smith based on his observations of the male genitalia [28]. Later, Mitchell [27] and Soroye and Bucks [34] classified *B. sandersoni* as a separate species from *B. vagans,* also based on differences in the malar length in spring-caught queens [28].

To reliably separate these species, previous authors sought to find morphologic characters that were consistent across a range of body sizes and castes to reliably differentiate species in the subgenus *Pyrobombus*. For example, Plowright and Pallett [28] proposed that spring-caught queen *B. sandersoni*, *B. vagans*, and *B. frigidus* specimens from a wide geographic range across Canada could be identified using several metrics including wing venation, and measurements of malar and antennal lengths. Following the methods in Richards [31], they verified that the length of malar spaces in spring-caught queen *B. vagans* did not overlap with either queen *B. sandersoni* or *B. frigidus*, but that the malar space did not significantly separate the queens of *B. frigidus* from *B. sandersoni*, concluding that the relationship between *B. sandersoni* and *B. frigidus* remained unclear and required further investigation [28]. They acknowledged that the longer malar length of *B. vagans* was the primary structural character used to separate the queens of *B. vagans* from *B. sandersoni*. Mitchell [27] compared the median length of the malar space in relation to the basal width of the mandible to differentiate the spring-caught queens of *B. sandersoni* from *B. vagans*. Although these morphometric characters are effective for distinguishing among spring-caught queens of *B. sandersoni* and *B. vagans*, none of these authors used these characters to separate worker specimens of these species. Size polymorphism in worker bumble bees can exhibit up to 10-fold variation in body mass within a single colony [35,36], and visual assessment of the malar length of smaller bodied bees without the aid of a microscope presents a greater challenge. This is important because workers are more abundant and active during the flight season than queens, and thus more likely to be encountered by field investigators and collected as specimens during community and population monitoring [37].

Earlier authors did not provide quantitative comparisons for morphologic features in worker castes, only in spring-caught queens. For example, Mitchell [27] described qualitative differences between *B. sandersoni* and *B. vagans* with respect to the malar character as “slightly greater”, “slightly shorter than”, or “nearly equal”, and Williams et al. [23] provide a similar qualitative assessment of the cheek length to width to distinguish between these species. Richards [31], in his work on the subgeneric divisions of *Bombus* Laterille, provided measurements of several morphometric characters using a micrometer scale (reticle) to provide measurements as units including those for absolute malar length and the proportions of antennal segments 3:4:5 in females, but he did not apply these findings to species within the subgenera. Although the descriptive morphological features used by previous authors provide a means for discriminating among these species, variability within species may make accurate determinations based on these characteristics difficult and have not been tested in workers [23,25].

To assess conservation assessments for species of bumble bees, and to monitor population status and trends, accurate identification is required. We undertook this study to establish reliable quantitative morphometric characters to distinguish among *B. sandersoni*, *B. vagans* and *B. perplexus*, using specimens of both queens and workers that were independently identified using DNA sequence data. We predicted that if these characters were useful for discriminating among species, then classifying species based on morphometrics would correspond to classifications based on DNA [37]. In addition, we assessed the accuracy of characters currently employed to separate these species, compare measurement repeatability within and between observers, and provide guidelines for accurate identification of this species complex for future studies.

## 2. Materials and Methods

### 2.1. Specimens

Specimens of worker and spring and fall-caught queen *B. vagans*, *B. sandersoni*, and *B. perplexus* were collected from 2010–2019 from the northern edge of their range in three states in the Midwest (Minnesota, Wisconsin, and Michigan) and three states in the northeast (New York, Massachusetts, and Maine) to represent regional diversity (see Supplemental Appendix A for complete specimen collection information). Initial species identifications were made on 216 specimens using taxonomic keys [23,27,38] and accompanying visual assessments of the ocular-malar area and hair color and patterns on the thorax and tergal segments. We did not include males in our analysis because they can be reliably identified using standard keys [23,27]. To confirm species identifications, we obtained DNA sequence data on 115 specimens haphazardly chosen from the 216 specimens to include individuals tentatively identified as one of the focal species based on visual observations, hair color pattern, including the presence of yellow hair on tergal segment 5 (T%), and individuals whose species assignment was not initially apparent (species information in Appendix A).

### 2.2. Determining Malar and Flagellar Segment Ratios

**Figure 1:**
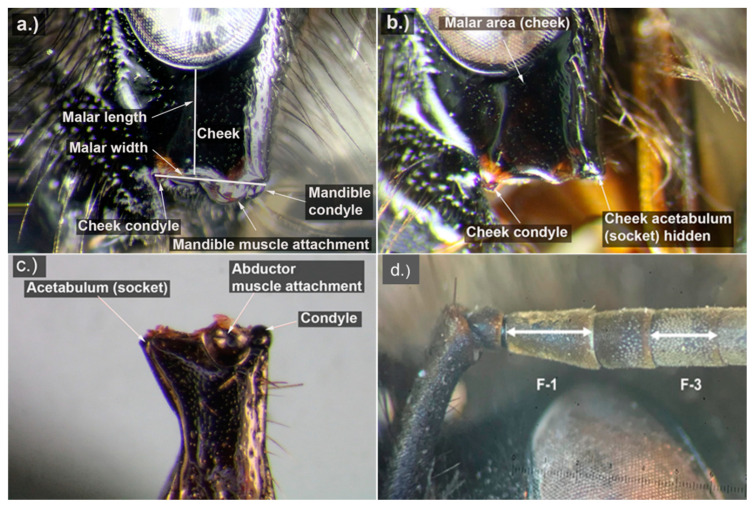
Images of a female *Bombus vagans* cheek and flagellar segments illustrating the measurement locations for the malar length, malar width, and lengths of flagellar segments 1 and 3. (**a**) Anatomy of the cheek. (**b**) Simplified image of the cheek with the mandible removed to show placement of the cheek condyle and cheek acetabulum. (**c**) Simplified image of the anatomy of the mandible. (**d**) Flagellar segments 1 and 3 illustrating where to make measurements to record length. Photos a-c by Dennis Johnson.

Using a reticle in the eyepiece of a stereomicroscope, we measured the malar length, width, and length of flagellar segments 1 and 3 on 216 specimens, including the 115 (44 *B. vagans*, 49 *B. sandersoni*, and 22 *B. perplexus*) that were subsequently chosen for DNA analysis. Following the anatomy in Michener [39] and Williams et al. [23], we measured the malar length (i.e., the shortest distance from the base of the eye to the edge of the cheek (Figure 1), and the malar width (i.e., the outside of the mandible condyle to the outside of the cheek condyle, assumed to be synonymous with the “basal width of the mandible” following Mitchell [27] (Figure 1a) on queens and workers to establish a range of MRL length to width ratios. To ensure consistency of measurements, we measured the malar length on both the vertical and horizontal axis to confirm that our measurements were constant.

To take malar width measurements, we used the cheek condyle to demark the medial edge of the mandible, because when the mandible of the specimen is closed, the small corner of the acetabulum is hidden by the lateral edge of the clypeus (Figure 1b,c). The cheek condyle aligns with the hidden edge of the mandible and therefore marks the edge of the mandible (Figure 1b). The malar length to width ratio is calculated as MRL = malar length/malar width. Next, we measured the horizontal lengths of flagellar segments 1 and 3. (Figure 1d) to establish a range of malar length to flagellar length ratios for flagellar segments 1 (MR1) and 3 (MR3) using the following calculations MR1 = malar length/flagellomer − 1, and MR3 = malar length/flagellomer − 3. Detailed instructions on how to measure the MRL, MR1, and MR3 ratios are provided in Appendix B.

To examine any possible effects from observer biases and experiences, each specimen was measured by two independent observers (Hereafter: Obs1 and Obs2). To determine the extent to which this methodology could be employed by a novice, specimens were measured by a student volunteer with < two months of experience identifying bees under a microscope (Hereafter: Obs3).

### 2.3. DNA Barcoding

To provide species-level identifications to determine the accuracy of existing keys and our MRL, MR1, and MR3 measurements, DNA was extracted from 115 specimens for which we had used existing keys and made visual identifications. From each specimen, the central-right leg was removed using sterilized forceps, legs were ground in a 1.5 mL microcentrifuge tube using a polypropylene pestle (USA Scientific, Inc., Ocala, FL, USA) and whole genomic DNA was isolated using the Omega Bio-tek E.Z.N.A.^®^ Tissue DNA Kit (Omega Bio-tek, Inc., Norcross, GA, USA). After isolation, a fragment of the mitochondrial locus cytochrome oxidase I (COI) was amplified using the protocol described in Hebert et al. [40]. Initial attempts to amplify the complete “barcode” fragment using the primers LepF1 and LepR1 as described in Hebert et al. [40], were unsuccessful, likely due to extractions that were performed from pinned specimens of varying ages. Therefore, we designed novel primers BombusF (5′-AGWCAYCCTGGAATATGAA-3′) and BombusR (5′-GTGGRAAAGCTATATCAGG-3′) to amplify ~150 base-pairs of the barcode fragment that was diagnostic among species. PCRs were conducted following conditions described in Smith-Freedman et al. [41], and DNA sequencing of both forward and reverse fragments was performed at the DNA Analysis Facility on Science Hill at Yale University. Raw sequence reads were edited in Geneious 11.1.2 (Biomaters Ltd., Auckland, New Zealand), and the forward and reverse reads assembled into a consensus sequence for each sample, and an alignment of all sequenced samples was then constructed in Geneious using the “Geneious Alignment Tool” with default parameters. Sequences were then assigned to haplotypes, and the relationships of haplotypes to each other were reconstructed using TCS v. 1.21 [42] with a 95% connection limit. Inter- and intraspecific percent differences between and among each species were then calculated in Geneious based on the species identifications from the TCS network analysis. DNA barcodes associated with all specimens are accessible on GenBank under the Accession numbers MT951454-MT951575, MT991562-MT991569 (Appendix A).

### 2.4. Statistical Analyses

To determine the accuracy of visual identifications (i.e., traditional identifications which are based on hair coloration and just visual observations of malar length) in comparison to DNA-confirmed identifications we performed Pearson’s correlation tests. To compare mean malar ratio measurements among the three species, we used a generalized linear model (GLM) that included the malar ratio measurement as our response (either MR1, MR3 or MRL), a fixed effect of species (identified by DNA barcoding), bee caste (queen or worker) and region (Midwest or Northeast). We also included an interaction term between species and caste and between species and region. We ran this model assuming a Gaussian error distribution and tested variable significance using analysis of variance (ANOVA). Non-significant interaction terms (*p* > 0.05) were removed, and the analysis rerun with the remaining fixed effects and interaction terms [43]. Pairwise differences among levels within our fixed effects with least-squares means were compared using the package ‘emmeans’ to acquire estimated means and Tukey-adjusted *p*-values [44].

To test the accuracy of the three malar ratio measurements for identifying species, we used a linear discriminant analysis (LDA) on queens and workers separately for *B. sandersoni* and *B. vagans. B. perplexus* was excluded from this analysis because we found that hair color, even on the less common light-colored specimens, contained a few scattered dark hairs on the thorax, and in most cases, light form specimens had light hair on the third tergal segment. Thus, malar ratio measurements were unnecessary for this statistical comparison. However, we present *B. perplexus* MRL, MR1, and MR3 ratio ranges to alert taxonomists to the possibility of rare, light-colored *B. perplexus* that may require additional characters to confirm identification.

We performed an LDA on 60% of our 115 DNA verified specimens to use as a training dataset and then predicted species identification on the remaining 40% of samples using MR1, MR3, and MRL measurements and validated the accuracy of our classifications using the species identifications from the DNA barcodes. We ran the LDA using the function ‘lda’ in package ‘MASS’ [45] and split the data using function ‘createDataPartition’ from package ‘caret’ [46]. To quantify the uncertainty in accuracy using three measurements, we performed a bootstrapping method to resample the dataset 999 times and determined the median ± SD of accuracy for the three measurements for both queens and workers separately.

We then predicted a range of values for each measurement beyond our measured individuals using the sample mean and SE. For each species/caste combo (e.g., *B. sandersoni* queens), we first bootstrapped the population mean and standard error by 500 sampled replicates using the function ‘boot’ from package ‘boot’ [47]. We then simulated a population of 10,000 bees from a normal distribution using the bootstrapped mean and SD and report the 99% quantile of the simulated distribution. All analyses were performed using the statistical environment R v. 3.5.1 [48].

Finally, we assessed the repeatability of malar ratio measurements by different observers. We compared measurements within and among observers using a Pearson’s correlation using the function ‘cor,test’ in R [48]. We first compared measurements by experienced Obs1 to measurements by both an experienced Ob2 and inexperienced Ob3. We also examined the correlation between standard deviation (SD) and the mean of repeated measurements within and between observers to determine if repeatability was associated with measurement size (e.g., observations become less reliable as individuals decrease in size).

## 3. Results

### 3.1. Malar Ratio Measurements & DNA Barcoding

We collected measurements for MRL, MR1, and MR3 from a total of 216 specimens and obtained DNA results from 115 specimens to confirm species identifications (49 *B. sandersoni*, 44 *B. vagans*, 22 *B. perplexus*). Without using quantified malar length to width or flagellar length ratios, our identifications based on existing keys and visual characteristics (e.g., hair color) were correct 70.4% of the time. For all worker and queens, the malar length was smaller than the malar width. Correlation between the three malar ratio measurements were strong (MR1 to MR3:R = 0.66, MR1 to MRL R = 0.75, MR3 to MRL:R = 0.77). For all species comparisons, we use measurements from only one observer (Obs1) to avoid pseudoreplication.

**Figure 2:**
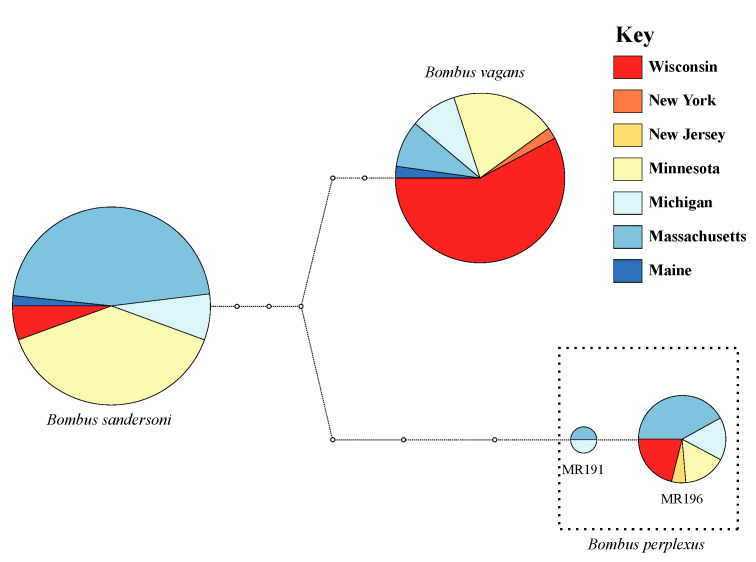
Statistical parsimony network highlighting the relationships of our three focal *Bombus* species. For each species, charts are drawn proportional to the number of sequenced individuals, and colored according to the geographic region from which individuals were collected. White circles are drawn to signify individual base pair differences between haplotypes.

Based on statistical parsimony reconstruction, individuals could be assigned to one of three groups representing individuals of (1) *B. sandersoni* (haplotype MR001), (2) *B. vagans* (haplotype MR003), and (3) *B. perplexus* (haplotypes MR191 and MR196 [one base-pair different between the two *B. perplexus* haplotypes]). The relationship of each group is presented in Figure 2.

Mirroring the network analyses, estimates of intra- and interspecific percent differences, found that individuals of *B. sandersoni* and *B. vagans* were 95.7% similar to each other (both species had 100% within species similarity), and that on average individuals of *B. perplexus* were 94.3% similar to individuals of both *B. sandersoni* and *B. vagans* (this species had 99.3% within species similarity due to the one-basepair difference between haplotype MR191 and MR196).

### 3.2. Comparing the Three Measurements among Species

**Figure 3:**
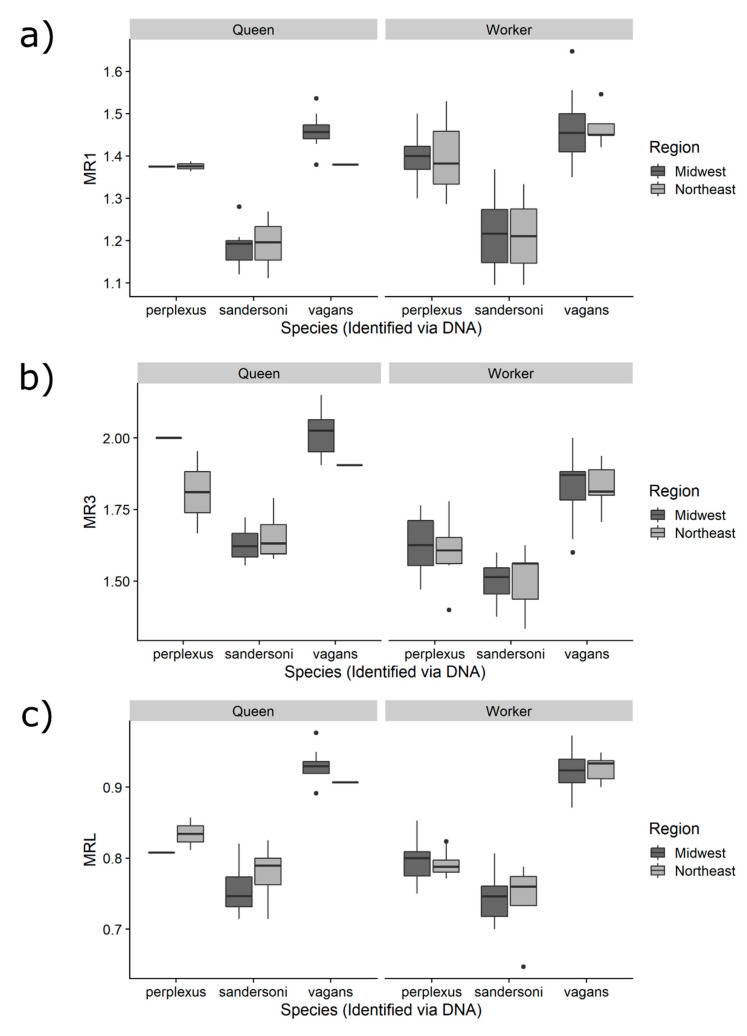
Comparison of malar ratios among the three *Bombus* species, caste, and between the regions for the following measurements: (**a**) MR1, (**b**) MR3 and (**c**) MRL.

For all three measurements, means were significantly different among the three species and between queens and workers (Table 1 and Table 2, Figure 3). However, differences between queens and workers varied among measurements. Queens had larger MR3 and MRL measurements compared to workers, but there was no difference in MR1 between queens and workers (Table 1 and Table 2, Figure 3). There were no significant differences in any measurement between the two geographic regions (Table 1) nor were there any significant interactions among species, castes, or region for any measurement (all *p* > 0.1).

### 3.3. Predictive Accuracy of the Three Measurements for Separating B. vagans and B. sandersoni

**Figure 4:**
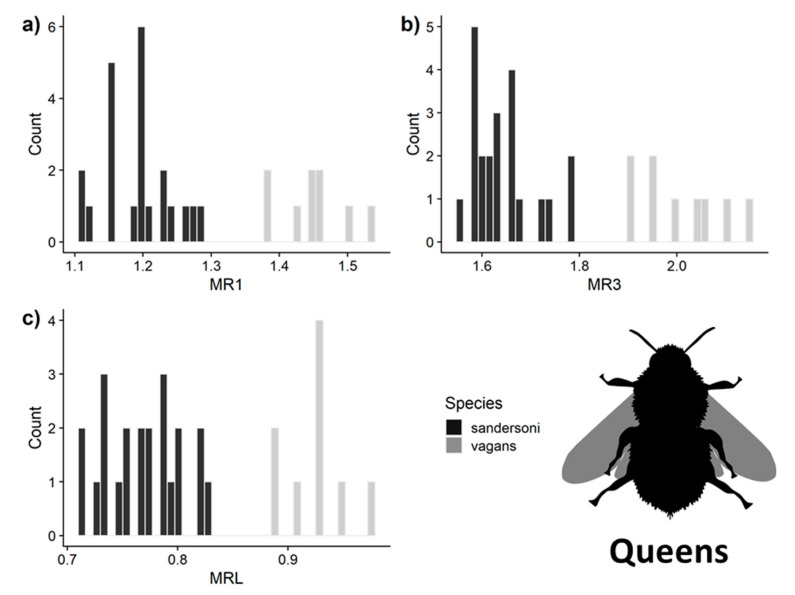
Distribution of malar ratio measurements of queens used in the linear discriminant analysis (LDA) for (**a**) malar length to flagellar segments 1 (MR1), (**b**) MR3 and (**c**) MRL. All three measurements had 100% accuracy for predicting species identities on subsampled datasets. Image from phylopic.org.

**Figure 5:**
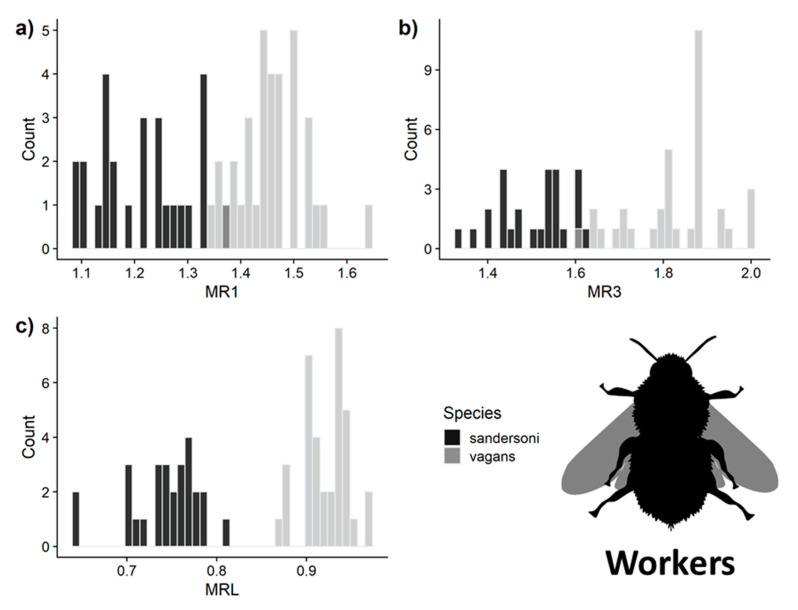
Distribution of malar ratio measurements of workers used in the LDA for (**a**) MR1, (**b**) MR3 and (**c**) MRL. Measurement accuracy ranged from 95–100%. Image from phylopic.org.

Using the measurements of queens, the LDA model predicted species identity with 100% accuracy for all three malar ratio measurements (Figure 4). For workers, MRL had 100% ± 0% SD and MR1 had 96% ± 5% SD accuracy, and MR3 had a 97% ± 4% SD accuracy (Figure 5). Only MRL had complete separation between *B. vagans* and *B. sandersoni* for queens and workers. However, using both MR1 and MR3 measurements to differentiate species resulted in 99% ± 0.01% SD accuracy. We report characteristics of the three species and the min and max of all measurements as well as the 99% quantile of our simulated distributions (Table 3).

### 3.4. Correlation within and between All Observers

Repeat measurements of MR1, MR3, and MRL were highly correlated within observers, as were measurements between both experienced observers (R > 0.9, Table 4). Correlations between experienced and inexperienced observers were also strong but slightly lower than ratios measured by experienced observers (R = 0.81compared to 0.85, Table 4). The standard deviation of measurements was small (range: 0 to 0.11), and for MR1 and MR3 there was no evidence of a relationship between the standard deviation of measurements and the mean in either measurement for both experienced and inexperienced observers (Table 4). There was a weak tendency for SD to decrease as mean increased for MRL (R-0.18, Table 4), suggesting this measurement may be more difficult to measure accurately when specimens are small.

## 4. Discussion

Accurate methods for bumble bee identifications are a critical component of any monitoring and conservation effort. Here, we present methods that allow researchers to discriminate among queens and workers of three cryptic eastern bumble bee species (*B. sandersoni*, *B. vagans*, and *B. perplexus*) with 100% accuracy in MRL ratios. We document that there was little variation among observers, even novices, in measurements, suggesting these methods, if properly employed, are robust with respect to measurement error. The quantification of defined ranges of these measurements presented for each species provides a replicable and timely tool for species identification given the current conservation interest in pollinator species and the prevalence of regional *Bombus* surveys to inform conservation best management practices.

When we used hair color or observations of the malar length as characters to separate *B. sandersoni*, *B. vagans*, and *B. perplexus* based on descriptions found in taxonomic sources e.g., [23,27,38] our rate of correct identification was far lower than the identification rate using our morphometric measurements (70% using visual descriptions vs 100% using MRL and 99%+ using both MR1 and MR3 together). Validated by DNA identification, we found visual characteristics, particularly hair color, could vary widely among individuals. For example, T5 in queens and worker *B. vagans* and *B. sandersoni* could be either all black or have widely varying amounts of yellow hair from few to many. Although Williams et al. [23] describes this variation, other sources such as Discoverlife.org suggest that *B. vagans* workers always have T5 black except in specimens collected in Newfoundland. In our specimens, the presence of yellow hairs on T5 was found in specimens collected in four different states in both the Midwest and Northeast regions of the United States. Further complicating hair color as a useful identification tool is preparation quality, such that hair color is often difficult to distinguish in *Bombus* specimens with matted hair, a not uncommon state of some *Bombus* specimens in collections. We found that *B. perplexus* can usually be identified by some of the following hair color patterns (1) the scutum has all light hair, or rarely with a few black hairs whereas *B. vagans and B. sandersoni* usually have many black hairs in this area, (2) the lower half of the pleura usually has dark hairs (common dark form), which the other two species do not, (3) the uncommon “light form” appears to have no dark hairs on the pleura, although often a few can be found low down around the legs, and (4) light form *B. perplexus* often have some yellow hairs on T3 which would not be the case for the other two species. While hair color can be useful to identify most *B. perplexus*, careful examination of malar ratio measurements is essential to confirm identification of light-form *B. perplexus*, and in some challenging cases, DNA confirmation may still be necessary. Additionally, while the malar ratios we present were calculated based on the examination of physical specimens of workers and queens, it is possible that they could be integrated into future community science projects (also called ‘citizen science’) if detailed photographs of the malar region and flagellar segments were taken providing a clear image that could be digitally measured with our methods.

The morphological similarity among these three species highlights the inherent challenges to accuracy in community science projects that do not collect specimens. Goulson et al. [49] point out that community science surveys are often limited by the taxonomic skills of the observers, particularly for bee species that are difficult or impossible to identify in the field because of cryptic coloration. MacPhail et al. [50] reported that 46.3% of *B. vagans*, 38.6% of *B. sandersoni*, and 86.4% of *B. perplexus* were correctly identified from photos of *Bombus* by project designated expert taxonomists, or they were placed into a “two-striped species group” when the photos were ambiguous. Richardson et al. [13] found that they could reliably make a species determination from 68% of photographs submitted by participants in the Vermont Bumble Bee Atlas. Suzuki et al. [51] in their survey of bumble bees in Japan found that they had high consistency of identification from photos that ranged from 95–97.7%. The higher success rate in Japan could be contributed to fewer species that exhibited similar hair patterns. However, none of these identifications were validated with DNA sequence data. For our three focal species with similar hair coloration, the use of photographs alone as a means for identification represents a cautionary tale.

Prior to this study, there were no established quantitative morphometric measurements ranges for workers of these three species. In his comprehensive paper “The Bombidae of the New World,” Franklin [25], included a chart of the range of malar spaces for spring-caught queen *B. vagans*, as lengths vs. the widths of the eye expressed in Filar micrometer spaces (divisions) and expressed them as ratios. Using spring-caught queen *B. sandersoni* and *B. vagans*, Plowright and Pallett [28] presented their results for the means and range lengths of malar spaces and the length of the malar space-to-length of the 3rd antennal segment. If we assume that their antennal segment three is equivalent to our flagellar segment 1, then their measurements were similar to our results for queen *B. vagans* but showed greater variation for queen *B. sandersoni*. There is some ambiguity in their results because the authors did not provide detailed instruction on where they made their malar and flagellar measurements, nor did they validate their specimen identifications using DNA sequence data. Based on our measurements for MRL, MR1, and MR3, and our DNA confirmed species identification, we have been able to provide a reliable range of values to differentiate *B. vagans* and *B. sandersoni*. However, and perhaps not unexpected given its name, our values for *B. perplexus* overlapped the other two species, though generally accurate identifications can be made based on observations of hair coloration combined with malar ratios.

Although the three species we discuss in this paper are considered species of least conservation concern by ICUN criteria at this time [52], we recognize that bumble bees that were once considered common can experience rapid declines in population size and range restrictions as witnessed in *Bombus affinis* Cresson, *B. terricola* Kirby, and *B. pensylvanica* Degeer; species now considered Critically Endangered or vulnerable by the IUCN SSC Bumblebee Specialist Group (https://bumblebeespecialistgroup.org/north-america/). Moreover, population status for *B. vagans* and *B. sandersoni* varies considerably over different regions. For example, Jacobson et al. [11] found a significant decline in *B. vagans* in New Hampshire over the past 150 years. Similarly, *B. vagans* was considered to be in decline in Canada in 2008, but then deemed stable in 2012 because of an increase in records despite a reduction in historical range size [7,53] and was considered a candidate for additional monitoring [54]. In Illinois, Grixti et al. [55] report that *B. vagans* was locally extirpated from its historic southern and northern ranges. In a review of historical changes in US bees with shared ecological traits, Bartomous et al. [5] found that *B. vagans* exhibited a decline but that *B. sandersoni* was stable. Franklin [56] considered *B. sandersoni* to be one of the most abundant species in its range, but it was rare in New Hampshire [11], and in Canada it was considered a candidate for immediate conservation concern [52] despite its status of Least Concern on the ICUN Red List [53]. Similarly, Richardson et al. [13] noted a 53% decline in *B. sandersoni* although a 266% increase in *B. vagans* abundance in in Vermont. *B. perplexus* appears to be increasing overall in abundance in the US and Canada [5,53]. Goldstein and Ascher [57] suspected that *B. sandersoni* may be under-reported on Martha’s Vineyard, MA because of its similar coloration with *B. vagans*, and to lesser degree, *B. perplexus*. Thus, conflicting reports of population trends in these cryptic species could be due in part to misclassification of specimens due to limitations in the diagnostic characteristics that were used to identify these species. This highlights our argument that accurate identifications are essential to accurately track population status and trends consistently across regions.

## 5. Conclusions

Around the globe, efforts are currently underway to examine changes in the distributions and abundance of pollinator species [17,58,59,60], with particular focus on the status of the native species [7,52,61,62,63,64]. However, for country and state-wide monitoring programs, or large-scale community science programs such as Bumble Bee Watch (https://www.bumblebeewatch.org/) and their associated conservation projects, providing accurate information on bumble bee population size and distribution depends on reliable identifications. The methods we present allow researchers to accurately discriminate among queens and workers of three cryptic species of bumble bee that had formerly posed a challenge to identify, particularly in worker specimens. The standardization of the MRL, MR1, and MR3 methods as we have defined them, and the range of values for each of the three species, provides an important tool to reliably identify species beyond visual characters such as hair coloration for *B. perplexus* and assessments of malar length in *B. vagans* and *B. sandersoni*. DNA analysis is an excellent tool to provide species identifications [65], but not everyone has access to DNA facilities or funding. In addition, care needs to be taken when interpreting DNA analyses (particularly for DNA barcoding projects) as results can often be misleading due to factors such as (but not limited to) the unintended amplification of non-target organisms (e.g., parasites, parasitoids, endosymbionts, etc.), the amplification of non-target loci (e.g., nuclear copies of mitochondrial DNA), and misidentifications in public databases (e.g., BOLD or GenBank). We are encouraged that the combination of DNA sequencing and morphological measurements was able to illuminate characters that can be used to accurately identify members of this confusing group of bumble bees, and we hope that as new technologies (such as Next-Generation sequencing) allow for high-throughput analyses of communities of organisms, that similar synergistic studies will be performed in other groups as well. The measurements we provide here to determine MRL, MR1, and MR3 values for *B. sandersoni*, *B. vagans*, and *B. perplexus* can be done with a minimum investment in equipment and time and provides highly accurate identification outcomes of these cryptic species required to inform effective monitoring and management decisions.

## Figures and Tables

**Table 1:** Results from generalized linear model (GLM), ANOVA and pairwise differences of fixed effects comparing the measurement means among the three species and between castes and regions. ^†^ Species comparisons are the Tukey’s HSD adjusted *p*-value.

Response	Variable	Levels	β ± SE	df	F	*p* ^†^
MR1	Species			2110	183.18	<0.0001
		*perplexus-sandersoni*	0.18 ± 0.02	110		<0.0001
		*perplexus-vagans*	−0.07 ± 0.02	110		0.0009
		*sandersoni-vagans*	−0.25 ± 0.01	110		<0.0001
	Caste	Worker	0.02 ± 0.01	1110	2.10	0.15
	Region	Northeast	0.00 ± 0.01	1110	0.01	0.94
MR3	Species			2110	121.26	<0.0001
		*perplexus-sandersoni*	0.14 ± 0.02	110		<0.0001
		*perplexus-vagans*	−0.20 ± 0.03	110		<0.0001
		*sandersoni-vagans*	−0.34 ± 0.02	110		<0.0001
	Caste	Worker	−0.17 ± 0.02	1110	67.99	<0.0001
	Region	Northeast	−0.00 ± 0.02	1110	0.05	0.83
MRL	Species			2110	362.45	<0.0001
		*perplexus-sandersoni*	0.05 ± 0.01	110		<0.0001
		*perplexus-vagans*	−0.13 ± 0.01	110		<0.0001
		*sandersoni-vagans*	−0.18 ± 0.01	110		<0.0001
	Caste	Worker	−0.02 ± 0.01	1110	9.55	0.003
	Region	Northeast	0.01 ± 0.01	1110	1.22	0.27

**Table 2:** Estimated means from the linear model for the three malar ratio measurements and among the three species for both workers and queens. Results are averaged over the two levels of region.

Species	Caste	MR1 (Mean ± SE, 95% CI)	MR3(Mean ± SE, 95% CI)	MRL (Mean ± SE, 95% CI)
*perplexus*	Worker	1.40 ± 0.02, 1.37–1.42	1.63 ± 0.02, 1.59–1.67	0.80 ± 0.01, 0.78–0.81
	Queen	1.38 ± 0.02, 1.34–1.41	1.79 ± 0.03, 1.74–1.85	0.82 ± 0.01, 0.80–0.83
*sandersoni*	Worker	1.21 ± 0.01, 1.19–1.24	1.49 ± 0.02, 1.46–1.53	0.75 ± 0.01, 0.74–0.76
	Queen	1.19 ± 0.01, 1.17–1.22	1.66 ± 0.02, 1.62–1.69	0.77 ± 0.01, 0.75–0.78
*vagans*	Worker	1.46 ± 0.01, 1.44–1.48	1.83 ± 0.02, 1.80–1.86	0.92 ± 0.01, 0.91–0.93
	Queen	1.44 ± 0.02, 1.41–1.47	2.00 ± 0.02, 1.95–2.04	0.94 ± 0.01, 0.93–0.96

**Table 3:** Characteristics, observed, and predicted measurement ranges ^†^ for the three *Bombus* species in this study.

Species	Caste	N	Mesipisternum Hair Color	Presence of Black Hairs on Scutum	Presence of Yellow Hairs on T5	MR1 ^†^	MR3 ^†^	MRL ^†^
*perplexus*	Worker	19	dark-light	none-few	none-few	1.29–1.53 (1.24–1.56)	1.40–1.78 (1.37–1.86)	0.75–0.85 (0.73–0.85)
*sandersoni*	Worker	27	light	few-many	none-few-many	1.10–1.37 (1.00–1.42)	1.33–1.63 (1.30–1.70)	0.65–0.81 (0.64–0.84)
*vagans*	Worker	35	light	many	none-few-many	1.35-1.65 (1.30–1.62)	1.60–2.00 (1.57–2.08)	0.87–0.97 (0.86–0.99)
*perplexus*	Queen	3	dark	none-few	none	1.36-1.39 (1.35–1.40)	1.67–2.00 (1.54–2.20)	0.81–0.86 (0.78–0.87)
*sandersoni*	Queen	22	light	few-many	none-few-many	1.11–1.28 (1.07–1.31)	1.56–1.79 (1.48–1.81)	0.71–0.83 (0.68–0.85)
*vagans*	Queen	9	light	many	none-few-many	1.38–1.54 (1.33–1.57)	1.90–2.15 (1.80–2.22)	0.89–0.98 (0.86–0.99)

**^†^** Minimum and maximum values taken from Observer 1. Values inside parentheses are the estimated 99% quantile from the bootstrapped mean.

**Table 4:** Correlations between replicate measurements within and between experienced and inexperienced observers. Inexperienced Obs3 did not make MRL measurements therefore comparisons are not included here.

Comparison	Measurement	R	t	df	*p*
Within Obs1	MR1	0.95	33.08	113	<0.001
	MR3	0.94	28.48	113	<0.001
	MRL	0.92	25.50	113	<0.001
Within Obs2	MR1	0.92	25.68	113	<0.001
	MR3	0.90	21.61	113	<0.001
	MRL	0.86	18.12	113	<0.001
Between experienced observers (Obs1 vs. Obs2)	MR1	0.92	24.91	113	<0.001
	MR3	0.91	22.72	113	<0.001
	MRL	0.93	26.55	113	<0.001
Between experienced and inexperienced observers (Obs1 vs. Obs3)	MR1	0.85	8.69	29	<0.001
	MR3	0.81	7.39	29	<0.001
	MRL	NA	NA	NA	NA
Between SD and the mean (Obs1 vs. Obs2)	MR1	0.02	0.20	113	0.84
	MR3	0.11	1.22	113	0.23
	MRL	−0.18	−1.90	113	0.06
Between SD and the mean (Obs1 vs. Obs3)	MR1	0.02	0.12	29	0.90
	MR3	−0.02	−0.09	29	0.93
	MRL	NA	NA	NA	NA

## Data Availability

Limited data presented in the manuscript; complete data and specimens available upon request.

## Data Deposition

Specimen and genetic data reported in this paper are included in Appendix A.

## Appendix A

**Table A1:** Project bumble bee specimens for determining ratios of MRL, MR1, and MR3 presented by project number, caste, collection date, collection state, latitude, longitude, verified DNA results, and GenBank Accession numbers.

Project ID Number	Caste	Coll. Date m/d/yr.	State Collected	Latitude	Longitude	DNA Results	GenBank Accession Numbers
MR_003	w	8/23/2018	WI	45.1385	−88.4728	vagans	MT951529
MR_004	w	7/25/2018	WI	45.2704	−88.3468	vagans	MT951530
MR_076	w	7/23/2018	WI	45.2002	−88.5869	vagans	MT951532
MR_077	w	7/26/2018	WI	45.2996	−88.3899	vagans	MT951533
MR_079	w	7/26/2018	WI	45.2996	−88.3899	vagans	MT951534
MR_080	w	7/23/2018	WI	45.2002	−88.5869	vagans	MT951535
MR_082	w	7/18/2018	WI	45.1682	−88.3119	vagans	MT951536
MR_084	w	7/9/2018	WI	45.3415	−88.4195	vagans	MT951537
MR_085	w	7/9/2018	WI	45.3198	−88.4079	vagans	MT951538
MR_091	w	7/23/2018	WI	45.3199	−88.4072	vagans	MT951539
MR_092	w	7/23/2018	WI	45.3199	−88.4072	vagans	MT951540
MR_094	w	7/18/2018	WI	45.1682	−88.3119	vagans	MT951541
MR_097	Q	6/8/2018	MI	46.5008	−90.0185	vagans	MT951542
MR_102	w	6/26/2014	NY	43.7356	−73.8508	vagans	MT951543
MR_105	w	8/15/2018	MI	46.5365	−89.0134	vagans	MT951544
MR_118	Q	7/25/2018	WI	45.2704	−88.3468	vagans	MT991562
MR_119	Q	8/30/2018	WI	45.9942	−88.4572	vagans	MT991563
MR_121	w	8/14/2018	MN	47.7686	−90.8927	vagans	MT951545
MR_124	w	7/25/2018	WI	45.2704	−88.3468	vagans	MT951546
MR_127	w	8/15/2018	MI	46.5759	−88.8877	vagans	MT951547
MR_133	w	8/14/2018	MN	47.7856	−90.8823	vagans	MT951548
MR_134	w	8/14/2018	MN	47.7856	−90.8823	vagans	MT951549
MR_136	w	8/14/2018	MN	47.7856	−90.8823	vagans	MT951550
MR_144	w	7/19/2018	MA	42.6806	−72.1117	vagans	MT951551
MR_146	w	7/18/2018	MA	42.6614	−72.1083	vagans	MT951552
MR_151	w	7/10/2018	MN	47.7697	−90.3107	vagans	MT951553
MR_154	w	7/2/2018	MN	47.4935	−91.9518	vagans	MT951554
MR_160	w	8/14/2018	MN	47.7686	−90.8927	vagans	MT951555
MR_165	w	8/14/2018	MN	47.7686	−90.8927	vagans	MT951556
MR_167	Q	7/23/2018	WI	45.2719	−88.6892	vagans	MT991564
MR_168	w	7/18/2018	WI	45.1682	−88.3119	vagans	MT951557
MR_180	w	7/11/2014	MA	42.5312	−72.3220	vagans	MT951558
MR_184	w	8/22/2018	WI	45.3215	−88.4050	vagans	MT951559
MR_185	Q	5/15/2018	WI	45.3215	−88.4050	vagans	MT951560
MR_186	w	8/8/2018	WI	46.0250	−88.8744	vagans	MT951561
MR_187	w	8/8/2018	WI	46.0250	−88.8744	vagans	MT951562
MR_199	Q	7/10/2018	WI	45.1690	−88.3338	vagans	MT991565
MR_200	Q	7/10/2018	WI	45.1690	−88.3338	vagans	MT991567
MR_204	w	8/7/2018	WI	45.8141	−88.6540	vagans	MT951563
MR_207	w	7/19/2018	WI	45.2951	−88.5143	vagans	MT951564
MR_212	w	8/21/2018	MI	46.3424	−89.4668	vagans	MT951565
MR_215	Q	7/10/2018	WI	45.1690	−88.3338	vagans	MT951566
MR_219	w	7/10/2015	MA	42.5225	−72.3233	vagans	MT951567
MR_220	Q	5/25/2014	ME	43.8978	−69.7676	vagans	MT991566
MR_001	w	7/11/2018	MA	42.3481	−72.2324	sandersoni	MT951476
MR_011	w	7/11/2018	MA	42.3481	−72.2324	sandersoni	MT951477
MR_013	w	7/11/2018	MA	42.3466	−72.2272	sandersoni	MT951478
MR_015	w	7/11/2018	MA	42.3446	−72.2275	sandersoni	MT951479
MR_017	w	7/11/2018	MA	42.3481	−72.2324	sandersoni	MT951480
MR_040	w	6/5/2018	MN	47.8405	−90.7564	sandersoni	MT951483
MR_042	w	6/8/2018	MN	47.5978	−90.8237	sandersoni	MT951484
MR_043	Q	6/5/2018	MN	47.7689	−90.8913	sandersoni	MT951485
MR_047	w	7/9/2018	MN	47.5977	−90.8242	sandersoni	MT951486
MR_048	w	7/10/2018	MN	47.7697	−90.3107	sandersoni	MT951487
MR_052	w	7/9/2018	MN	47.5977	−90.8242	sandersoni	MT951488
MR_053	w	7/2/2018	MN	47.4935	−91.9518	sandersoni	MT951489
MR_055	w	7/9/2018	MN	47.5977	−90.8242	sandersoni	MT951490
MR_057	w	7/9/2018	MN	47.5977	−90.8242	sandersoni	MT951491
MR_058	w	7/9/2018	MN	47.5977	−90.8242	sandersoni	MT951492
MR_060	w	7/10/2018	MN	47.7697	−90.3107	sandersoni	MT951493
MR_061	w	7/10/2018	MN	47.7697	−90.3107	sandersoni	MT951494
MR_062	w	7/10/2018	MN	47.7697	−90.3107	sandersoni	MT951495
MR_065	w	7/10/2018	MN	47.7697	−90.3107	sandersoni	MT951496
MR_067	Q	8/13/2018	MN	47.5977	−90.8242	sandersoni	MT951497
MR_087	w	7/17/2014	MA	42.5020	−72.3690	sandersoni	MT951499
MR_088	Q	5/6/2014	MA	42.4310	−72.2500	sandersoni	MT951500
MR_095	Q	7/23/2018	MI	46.2213	−86.6676	sandersoni	MT951501
MR_099	Q	5/23/2018	MN	48.0534	−90.0562	sandersoni	MT951503
MR_103	w	6/9/2010	ME	44.3000	−68.3500	sandersoni	MT951504
MR_108	Q	5/5/2014	MA	42.4182	−72.2535	sandersoni	MT951505
MR_109	Q	5/5/2014	MA	42.4187	−72.2439	sandersoni	MT951506
MR_113	Q	5/5/2014	MA	42.4312	−72.2501	sandersoni	MT951507
MR_139	Q	6/12/2018	MA	42.3426	−72.2357	sandersoni	MT951508
MR_142	Q	7/11/2018	MA	42.3504	−72.2274	sandersoni	MT951509
MR_143	w	7/18/2018	MA	42.6590	−72.1068	sandersoni	MT951510
MR_178	Q	5/5/2014	MA	42.4141	−72.2546	sandersoni	MT951511
MR_179	Q	5/5/2014	MA	42.4141	−72.2546	sandersoni	MT951512
MR_181	w	7/9/2014	MA	42.4407	−72.2490	sandersoni	MT951513
MR_214	w	6/20/2018	MI	46.3424	−89.4668	sandersoni	MT951514
MR_216	Q	5/6/2015	MA	42.4187	−72.2439	sandersoni	MT951515
MR_217	w	7/6/2015	MA	42.4188	−72.2438	sandersoni	MT951516
MR_218	w	7/16/2015	MA	42.5225	−72.3233	sandersoni	MT951517
MR_221	Q	5/3/2015	MA	42.5005	−72.2691	sandersoni	MT951518
MR_224	w	6/20/2018	MI	46.3424	−89.4668	sandersoni	MT951519
MR_225	w	6/6/2017	MN	47.7964	−90.9315	sandersoni	MT951520
MR_229	Q	5/2/2015	MA	42.5312	−72.3219	sandersoni	MT951521
MR_231	w	6/6/2017	WI	47.7660	−88.9701	sandersoni	MT951522
MR_232	w	6/5/2017	WI	45.7384	−88.5829	sandersoni	MT951523
MR_235	Q	5/2/2015	MA	42.5022	−72.3697	sandersoni	MT951524
MR_237	Q	6/2/2018	WI	45.1311	−88.3738	sandersoni	MT991568
MR_238	Q	6/6/2017	MN	47.2935	−91.9503	sandersoni	MT951525
MR_239	Q	6/6/2017	MN	47.2935	−91.9503	sandersoni	MT951526
MR_241	Q	6/6/2017	MN	47.2935	−91.9503	sandersoni	MT951527
MR_009	w	6/20/2018	WI	45.9363	−88.9506	perplexus	MT951454
MR_039	w	6/7/2017	MI	44.1351	−85.9340	perplexus	MT951455
MR_050	w	8/23/2018	WI	45.1385	−88.4728	perplexus	MT951456
MR_071	w	8/13/2018	MN	47.7856	−90.8823	perplexus	MT951457
MR_115	w	5/24/2012	MA	42.3063	−72.6946	perplexus	MT951458
MR_117	w	5/24/2012	MA	42.3063	−72.6946	perplexus	MT951459
MR_130	w	6/20/2018	WI	45.9363	−88.9506	perplexus	MT951460
MR_189	w	6/20/2018	WI	45.9363	−88.9506	perplexus	MT951461
MR_190	w	8/13/2018	MN	47.7695	−90.3098	perplexus	MT951462
MR_191	w	8/8/2018	MI	46.2715	−89.4930	perplexus	MT951463
MR_192	w	8/8/2018	MI	46.2715	−89.4930	perplexus	MT951464
MR_193	w	6/5/2018	MN	47.7689	−90.8913	perplexus	MT951465
MR_194	Q	8/18/2018	MI	46.19618	−89.1594	perplexus	MT951466
MR_195	w	8/15/2017	MI	46.3375	−89.4753	perplexus	MT951467
MR_197	w	7/11/2018	MA	42.3399	−72.2330	perplexus	MT951469
MR_198	w	7/16/2015	MA	42.4940	−72.2706	perplexus	MT951470
MR_247	w	7/1/2019	MA	42.5824	−72.5301	perplexus	MT951471
MR_248	w	7/3/2019	MA	42.5824	−72.5301	perplexus	MT951472
MR_249	w	7/3/2019	MA	42.5824	−72.5301	perplexus	MT951473
MR_250	w	7/3/2019	MA	42.5824	−72.5301	perplexus	MT951474
MR_251	Q	7/3/2019	MA	42.5824	−72.5301	perplexus	MT951475
MR_255	Q	4/25/2019	MA	42.3928	−72.5309	perplexus	MT991569

## Appendix B

Directions on how to measure the malar length and width (MRL) (technique developed by Dennis E. Johnson), and how to measure the lengths of flagellar segments 1 and 3 to find MR1, and MR3 ratios adapted from Plowright and Pallett (1978).

### Appendix B.1. Equipment Required

1. A stereo microscope, preferably with a 45–50× zoom capability.
2. A 10× eyepiece with a reticle for measuring with at least one axis with 100 divisions in increments of 10 is preferred but other reticles work as long as the scale is large enough to measure the malar length and width.
3. Reticles can be acquired for most, but not all, microscope eyepieces. Reticles regularly require a diopter adjustment to focus the reticle properly to ensure that the image of the reticle divisions are sharp and clear. It is helpful to first perform this focus exercise on the microscope stage without a specimen. The diopter adjustment compensates for differences between the observers’ eyes so it should be re-adjusted for each user to compensate for variability among observers. Instructions for diopter adjustments can be found online for microscopes and binoculars.
4. Specimens must be oriented so that the body part being measured is oriented perpendicular to the axis of observation (e.g, place the specimen in a flat, horizontal position). A specimen manipulator allows for a pinned specimen to be viewed from multiple angles while remaining in a central viewing area under the microscope while allowing the viewer to maintain relative focus while twisting or turning a specimen to show all sides. A large cork can also be used, but it requires more patience to properly orient the specimen for measurement on a flat plane.
5. To make the measurements, either rotate eyepiece to orient the reticle in a horizontal or vertical position or move the specimen. Try both methods to see which works best. Make one or more measurements to confirm that they are the same value.
6. It is extremely important not to change the zoom between measurements. The specimen can be moved, and the focus can be adjusted between the length and width measurements, but do not change the zoom power. Each time you zoom up or down in power, the actual magnification level may be slightly different. Changing the zoom level between measurements means that you risk making your measurements at different magnification levels and the resulting ratios will be incorrect. The power of the zoom used for making measurements is not important as long as both measurements are made at the same magnification.
7. Take care to ensure that both ends of the of the body part to be measured are in focus.
8. Good lighting is important in making accurate determinations. A cool white LED ring lamp works well.
9. Be careful to make sure that you are not 5 or 10 divisions off when reading the reticle measurement. This is a common error.

### Appendix B.2. How to Measure the MRL (Malar Length to Width)

1. Two measurements must be made to determine the malar ratio (MRL), i.e., the malar length and the malar width as shown in Figure 1a. The measurements of the malar length and width are made with the reticle and recorded as the number of reticle divisions. The malar length to width ratio is calculated using the following equation.
  MRL = malar length/malar width
2. Partial divisions are not estimated, rather the measurement is taken as the number closest to the endpoint.
3. The malar length is measured as the shortest distance from the base of the eye to the edge of the cheek using the definition from Williams et.al. [23]. The malar width is defined as the measurement from the outside of the mandible condyle to the outside of the cheek condyle (Figure 1a). This is equivalent to “cheek breadth” [23] or to the “basal width of the mandible” in Mitchell [27]. See Figure 1b,c for separate views of the cheek and mandible.
4. Running a pin tip along the bottom edge of the cheek (away from the eye) helps to locate the bottom end point to make the measurement.
5. We suggest measuring the length of the mandible at least twice, once on the horizontal axis and again on the vertical axis, to ensure that these values are equal.

### Appendix B.3. How to Measure the MR1 and MR3 (Malar Length to Flagellar Segment 1 and Malar Length to Flagellar Segment 3 Length)

1. Measure the malar length as described above for MRL measurements.
2. Measure the length of flagellomer-1 along the horizontal axis. Place the zero end of the reticle where the textured part of the segment begins (Figure 1d).
3. Measure flagellomer-3 on the horizontal axis (Figure 1d).
4. Calculations for malar length to flagellar segments 1 and 3 ratios are as follows
  MR1 = malar length/flagellomer − 1
  MR3 = malar length/flagellomer − 3
